# Mapping the genomic landscape of two key human gut microbial species: *Bifidobacterium infantis* and *Bifidobacterium longum*

**DOI:** 10.20517/mrr.2026.15

**Published:** 2026-05-09

**Authors:** Marco Ventura

**Affiliations:** ^1^Laboratory of Probiogenomics, Department of Chemistry, Life Sciences and Environmental Sustainability, University of Parma, Parma 43124, Italy.; ^2^Microbiome Research Hub, University of Parma, Parma 43124, Italy.

This article^[1]^ provides a comprehensive, large-scale genomic characterization of two key bacterial taxa inhabiting the infant gut, namely *Bifidobacterium longum* subsp. *infantis *(*B. longum *subsp. *infantis*) and *Bifidobacterium longum* subsp. *longum* (*B. longum *subsp. *longum*). It aims to understand how strain-level genetic differences translate into functional variation that shapes microbial ecology, host interactions, and ultimately host health, thereby enabling a more rational and predictive approach to designing health-promoting microorganisms. A key biological insight from this work^[1]^ is the extensive genomic heterogeneity within both *B. longum *subsp. *infantis* and *B. longum *subsp. *longum*, such that strains within the same species can differ substantially in gene content, metabolic pathways, and ecological fitness traits. This finding challenges the long-standing assumption that species-level classification is sufficient to infer probiotic function and instead emphasizes that biological activity must be interpreted at the strain level. This variability is most evident in genomic regions involved in glycan metabolism, particularly those associated with the utilization of human milk oligosaccharides (HMOs). These are complex carbohydrates abundant in human breast milk that constitute a key class of prebiotic compounds. The chromosomal sequences of *B. longum *subsp. *infantis* strains typically encompass large, highly specialized HMO-utilization gene clusters, enabling efficient internalization and degradation of these glycans. In contrast, *B. longum* subsp. *longum* strains often exhibit a more limited, fragmented, or variable capacity for HMO metabolism, indicating more diverse ecological strategies that are not uniformly optimized for the breastfed environment^[2]^. This finding has important biological implications, as it provides a mechanistic explanation for the frequent dominance of *B. longum *subsp.* infantis* in the microbiota of breastfed infants and supports the notion that diet, particularly the composition of human milk, plays a major role in shaping early-life microbial communities^[3,4]^. This taxon is rarely detected in the infant gut microbiota of industrialized populations but is more frequently observed in non-industrialized populations^[5]^.

At the same time, this study highlights that HMO metabolism alone is insufficient to explain colonization success, identifying additional genomic loci associated with carbohydrate transport systems, adhesion factors that modulate interactions with the intestinal epithelium, resistance to environmental stressors such as low pH or bile salts, and regulatory systems that enable bacterial responses to changing gut conditions. These findings reinforce the view that microbial colonization of the human gut is a complex, multifactorial process requiring the integration of metabolic specialization, ecological resilience, and host interaction capabilities^[6]^. Another important insight is the presence of functional trade-offs across strains, whereby some lineages are highly specialized for efficient HMO metabolism but may have reduced plasticity in utilizing alternative carbon sources, whereas others possess broader metabolic repertoires that enable persistence in more variable or HMO-poor environments^[7]^. These data suggest an ecological niche partitioning within the infant gut and imply that microbial diversity may be maintained through complementary functional strategies rather than competitive exclusion. This observation has direct implications for probiotic design, arguing against the use of a single “optimal” strain and instead supporting the development of multi-strain consortia capable of performing a broader range of functions and adapting to diverse ecological contexts^[8]^. The successful integration of multi-strain consortia, particularly those including allochthonous strains, remains a key challenge in the co-evolving early-life microbiota; therefore, complementary strategies, such as the provision of selective substrates, may be required to facilitate their establishment and long-term functional persistence in the human gut. This article also provides evidence of a tight co-evolutionary relationship between the human host and its early-life microbiota, as the genomic characteristics of *B. longum *subsp. *infantis* appear finely tuned to the structural diversity of HMOs. This suggests that human milk composition has evolved, at least in part, to selectively nourish beneficial microorganisms that, in turn, contribute to infant health through the production of metabolites such as short-chain fatty acids, modulation of immune development, and provision of colonization resistance against pathogens. From a systems biology perspective, this study represents a shift toward integrating genomic data with ecological and functional interpretations. This integration enables the prediction of strain behavior based on genomic features, thereby moving the field toward a more mechanistic understanding of microbiome assembly^[1]^. Notably, the authors translate these insights into a framework for rational probiotic design, in which strains can be selected based on defined genomic criteria, such as the presence of complete HMO-utilization loci, adhesion factors, and stress-response genes, with the goal of improving colonization efficiency and functional output in the infant gut. However, despite these advances, several limitations should be considered when interpreting the biological significance of the data presented in this study. In this context, it is worth noting that this work relies heavily on genomic inference, and the presence of specific genes does not necessarily guarantee their expression or activity under *in vivo* conditions, as gene regulation is influenced by environmental factors, host conditions, and interactions with other microorganisms. Furthermore, the infant gut is a highly complex and dynamic ecosystem, and a primary focus on *Bifidobacterium* species may overlook important interactions with other microbial taxa, as well as with bacteriophages, fungi, and the host immune system, all of which influence colonization dynamics and functional outcomes. Moreover, although the study provides strong predictions regarding which strains are likely to colonize effectively and utilize specific substrates, it does not directly establish causal links between these microbial traits and long-term health outcomes in infants - such as immune maturation, metabolic programming, or disease risk - thereby leaving an important gap between genomic potential and clinical relevance. In addition, evolutionary interpretations, while compelling, are largely based on genomic correlations and would benefit from experimental validation to demonstrate direct adaptive relationships between host milk composition and microbial gene content. Despite these limitations, the study makes a significant conceptual contribution by redefining health-promoting microorganisms not as generic species but as collections of strains with distinct and measurable functional capacities, highlighting the importance of host-microbe co-evolution in shaping early-life microbiomes, and providing a roadmap for development of next-generation probiotics tailored to specific ecological and physiological contexts. Notably, these data provide new and compelling evidence for the presence of members of the *B. longum* subsp. *infantis* taxon within the infant gut microbiome. Until now, this subspecies has only rarely been detected in the human gut, leading to ongoing uncertainty regarding its actual prevalence and functional relevance within the microbial community. Consequently, questions remain regarding its contribution to the overall structure and dynamics of the gut microbiota.

The findings presented here help address these uncertainties by providing new insights into the ecological role of *B. longum* subsp. *infantis*. In particular, they offer new perspectives on its potential biological functions within the infant gut ecosystem, as well as on its interactions with other microbial members. These results suggest that this subspecies may play a more significant and previously underappreciated role in shaping the composition and activity of the infant gut microbiota than has been previously recognized.

Overall, this work advances our understanding of how genomic diversity within key commensal bacteria translates into ecological behaviors and host interactions. It also underscores the importance of diet as a primary driver of microbiome assembly in the early life, and establishes a foundation for transitioning from descriptive microbiome research to predictive and intervention-oriented approaches, while emphasizing the need for integrative studies that combine genomics with experimental validation and clinical investigation to fully realize the potential of microbiome-based therapies.

## DECLARATIONS

### Copyright

© The Author(s) 2026.
